# A Whole Food Plant-Based Approach to Ulcerative Colitis; A Case Series

**DOI:** 10.1177/15598276231213325

**Published:** 2023-11-09

**Authors:** Despina Marselou, Shireen Kassam

**Affiliations:** 1 Clinical Dietitian and Nutritionist, Athens, Greece (DM); 2 4616 King’s College London and King’s College Hospital, London, UK (SK); 3 University of Winchester, Hampshire, UK (SK)

**Keywords:** diet, nutrition, lifestyle, plant-based, ulcerative colitis

## Abstract

Inflammatory bowel disease (IBD) is a relapsing and remitting condition that requires continuous treatment to reduce the risk of relapse. Alongside genetic factors, diet and lifestyle factors are heavily implicated in the pathogenesis of the disease, with diets high in meat and ultra-processed foods and low in fibre-rich plant foods thought to be central to the disease process. There is considerable interest in using dietary interventions to prevent, treat and IBD, with the hope that this can limit and, in some cases, even eliminate the use of pharmaceutical interventions. A whole food plant-based diet (WFPBD) is an attractive option given its emphasis on foods that promote gut health and reduce inflammation and the avoidance of foods that are associated with dysbiosis and inflammation. Here we describe 3 case histories of patients with ulcerative colitis and the successful use of a WFPBD for remission induction and maintenance with over 2 years of follow-up.

‘It is clear that most patients with IBD assume diet is an important factor in the development and treatment of the disease’.

## Introduction

Inflammatory bowel disease (IBD), both ulcerative colitis (UC) and Crohn’s disease, has been termed a ‘slow-motion’ epidemic, with Western and recently industrialised countries seeing a continued rise in incidence.^1^ Alongside genetic factors, it is thought that this trend is predominantly driven by environmental and lifestyle exposures, with dietary factors heavily implicated.^2^ UC is a relapsing, remitting, chronic inflammatory disorder that affects the colon and rectum.^3^ Dietary factors implicated in disease development are a Western-style diet pattern, high in meat and ultra-processed foods and low in fibre-rich plant foods, in part due to the adverse effects on the gut microbiome.^4-7^ In an analysis of 6 study cohorts from the US and Europe, adherence to healthy lifestyle behaviours (healthy weight, not smoking, regular physical activity and eating fruit and vegetables and sufficient fibre) was shown to have the potential to prevent around 50% of cases of UC.^8^ With this knowledge, there has been considerable interest in using diet as a treatment for UC, alone or in combination with established pharmaceutical medications.^9^ In particular, a whole food plant-based diet (WFPBD) has shown promise, given its focus on health-promoting foods that support a healthier gut microbiome and avoidance or limited consumption of the foods implicated in the development of UC.^7,10,11^ Here we present 3 cases of UC successfully treated with a WFPBD in a single dietetic clinic in Greece with at least 2 years of follow-up. For each case, the dietary pattern before and after adopting a WFPBD is summarised in Table 1.

**Table 1.** Dietary Pattern Before and After the WFPB Intervention.

	Usual Diet Before Intervention	Whole Food Plant-Based Diet
Case 1		
Grains	White pasta (twice a week) white rice (twice a week), white bread, white crackers (daily)	Buckwheat, millet, quinoa (daily), rice (once per week), wholemeal pasta and wholemeal bread (twice per week)
Beans, pulses	A variety (lentils, giant beans, black eyed peas, mung beans) once or twice per week	Red lentils, brown lentils, chickpeas, giant beans, mung beans (small portions e.g. 3 tablespoons daily), tofu twice per week
Vegetables	Green leafy vegetables (daily), okra, green beans, aubergines, peas once or twice per week	Broccoli, cauliflower carrots, sweet potato, potato, aubergine, tomato, mushrooms, peas, red bell peppers, green leafy veggies (a variety of 3 chosen vegetables daily)
Fruit	Banana every other day – rarely other fruits (every 3 weeks)	Banana (daily) strawberries (every other day), blueberries (daily), pineapple (daily), avocado (every other day), green and red apple (daily)
Nuts and seeds	None	Peanut butter, pumpkin seeds, linseeds or sunflower seeds (4-5 times per week)
Meat	Chicken, 3 times per week, red meat, twice per week, fish every other week	None
Dairy	Yoghurt and milk rarely (once every month)	None
Processed foods	Refined oil, sugar, butter, chocolate, sweets made with eggs and butter, fried foods, meat-based take-aways 3-4 times per week	Vegan cheese (twice per week), plant-based milk (daily)
Case 2		
Grains	White pasta, white rice daily	Millet/quinoa (daily), rice (twice per week), wholemeal pasta (once per week), wholemeal bread daily
Beans, pulses	None	Red lentils, brown lentils and then adding in chickpeas, giant beans, mung beans (small portions introduced gradually and on a daily basis and within a month whole portions combined with quinoa three times per week), tofu twice per week
Vegetables	Lettuce and tomato mainly on a daily basis	Αrtichoke, broccoli spinach, leek, carrots, sweet potato, potato, aubergine, tomato, mushrooms, peas, onions, red bell peppers, green leafy vegetables (daily with a variety of 3 chosen vegetables)
Fruit	Banana and apple daily	Banana, strawberries, blueberries, pineapple, avocado, green and red apple daily
Nuts and seeds	Walnuts and almonds daily	Peanut butter, pumpkin seeds, linseeds, hemps seeds (daily, 1 small portion)
Meat	Chicken daily, red meat twice per week	None
Dairy, eggs	Yoghurt rarely, milk daily	None
Processed foods	Refined oils, sugar, butter, chocolate, sweets made with eggs and butter, fried foods, meat-based take-aways 3-4 times per week	Vegan cheese (3 times per week), plant-based milk (daily) and vegan recipes from vegan blogs (once per week)
Case 3		
Grains	White pasta (3 times per week), white rice (daily), white bread (daily)	Millet/quinoa (daily), rice (twice per week), wholemeal pasta, wholemeal bread, oats (daily)
Beans, pulses	None	Red lentils, brown lentils and then adding in chickpeas, giant beans, mung beans (small portions introduced gradually and within a month whole portions combined with quinoa)
Vegetables	Tomatoes, cucumber, carrot, potato daily	Broccoli, leek, carrots, sweet potato, potato, aubergine, tomato, red bell peppers, green leafy vegetables (rocket, spinach)
Fruit	Banana every other day	Banana, blueberries, avocado, melon, nectarine, watermelon daily
Nuts and seeds	None	Peanut butter, almond butter or chia seeds daily
Meat	Chicken twice per week, red meat once a week, fish once a week, turkey and ham daily	None
Dairy, eggs	Yoghurt and milk rarely, 2 portions of cheese daily, 2 portions of eggs alternate days, butter daily	None
Processed foods	None	Vegan cheese (twice per week) and plant-based milk daily

## Case 1

A 35-year-old female (non-smoker) presented to the dietetic clinic in March 2021 after the birth of her second child with a diagnosis of UC. Her symptoms include bloody stools with mucus, excessive gas and severe constipation. A colonoscopy with biopsies confirmed UC with moderately active disease extending 30 cm from the anal verge. She had experienced similar symptoms during her first pregnancy, aged 31, which continued for 13 months, but this had not been extensively investigated. Her past medical history included recurrent tonsillitis as a child with repeated courses of antibiotics until a tonsillectomy was performed aged 18 years. At age 27 years she developed erythema nodosum, which was successfully treated with antibiotics, suggesting an infective aetiology.

The patients’ medical team had initiated treatment with mesalazine, but this had worsened symptoms with bloody diarrhoea and abdominal pain. Mesalazine was therefore stopped, and the patient opted to try a dietary approach.

The patient's baseline diet did include a variety of plant foods, but also meat or fish on most days, refined grains and processed foods. The dietary recommendations were based on a WFPBD (see Table 1). After one week, the patient complained of severe bloating and worsening constipation. This led to a 6-week recommendation of limiting foods high in FODMAPS (Fermentable, Oligo-saccharides, Di-saccharides, Mono-saccharides and Polyols) and increasing fibre intake more gradually, along with increasing hydration with the addition of soups. Within 3 weeks, there were improvements in her symptoms, with further continued improvement and subsequent resolution of all symptoms over the next few months. The repeat colonoscopy in Sept 2021 showed no inflammation in the sigmoid colon and significant improvement in the rectum, with evidence of healing ulcers, scar tissue and some redness extending up to 10 cm.

After the initial symptomatic response to this dietary approach, the patient introduced some animal foods back into her diet, such as meat, fish and eggs, which was associated with a relapse during her third pregnancy. The patient therefore reverted to a mostly WFPBD with occasional consumption of fish and chicken and was symptom-free at the last follow-up in June 2023.

## Case 2

A 35-year-old male presented to the dietetic clinic in April 2021 with a diagnosis of UC. His symptoms included bloody stools with mucus, excess gas and diarrhoea. He was receiving treatment with oral mesalazine. His medical history included a diagnosis of irritable bowel syndrome in 2017 when he received antibiotics to treat diarrhoea. Colonoscopy and biopsies performed in February 2021 showed extensive and severe inflammation of the colon, crypt abscesses and haemorrhage.

The patient had a very active lifestyle with regular exercise, including bodybuilding, and he had been taking multiple nutritional supplements, including branched-chain amino acids, creatine, protein and high-dose multivitamins. Prior to his diagnosis, the patient had stopped smoking cigarettes and was now smoking e-cigarettes. His baseline diet was high in animal protein, refined carbohydrates and frequent consumption of takeaway meals.

He commenced a WFPB diet under dietetic supervision. In the initial 3 months, he lost some weight (3kg), which the patient did not want, so higher calorie plant meals were recommended, including avocado, banana and peanut butter smoothies and increased portions of quinoa, seeds, pulses and sweet potato. A repeat colonoscopy in November 2021 was normal, and his clinical team advised him to stop mesalazine. At the last follow-up in June 2023, the patient continued to be on a 100% WFPB diet, with no relapses of UC and also reported improvement in his athletic performance.

## Case 3

A 41-year-old male presented to the dietetic clinic in October 2020 with a diagnosis of UC, recurrent relapses every 6 months, persistent inflammation present on annual colonoscopies and elevated calprotectin level (206 ug/g). His initial diagnosis was in 2017, with symptoms of bloody diarrhoea, mucus and excess gas. He was on oral mesalazine along with daily enemas. His past medical history included severe migraines since childhood, and he used non-steroidal anti-inflammatory drugs for this daily. He was a non-smoker but did report that his job as a truck driver was stressful.

His usual diet was heavy in meat (lamb and pork). His physician had recommended a low-fibre diet, but this had not helped his symptoms, and hence he had decided to seek further dietetic support.

He adopted a fully WFPBD under dietetic support. Within a few weeks, he reported improvement in symptoms, a reduction in mesalazine dose from 6 times per day to 4 and no further need for enemas. His repeat colonoscopy in April 2021 showed complete remission for the first time in 3 years. On review in February 2022, the patient reported a relapse of symptoms with blood diarrhoea and a rise in calprotectin levels to 345 ug/g. He reported going through a stressful life event related to his family situation, and he had also reintroduced meat into his diet. Following the review, he reverted back to a mostly WFPBD with occasional consumption of fish (once a month) and chicken (once or twice per month), introduced exercise into his routine and has had a further remission of symptoms, which has been maintained at his last follow-up in June 2023.

## General Recommendations for All Three Cases

All patients with IBD receive a personalised nutrition plan after considering their baseline diet, degree of symptoms and medical history. They receive general advice on choosing meals outside of the home and meal timings, such as finishing their final meal of the day at least 3 hours before sleep. All patients receive information on the benefits of good sleep, along with tips for improving sleep hygiene, and a recommendation to incorporate guided meditation into their daily routine. All patients are advised to supplement with vitamin B12. Vitamin D supplementation is only prescribed for those will low blood levels. The use of iodised salt is widespread in Greece. In general, whole nut consumption is limited in favour of nut butters due to the personal experience of the treating dietitian who finds that people with IBD do not tolerate them well. A general emphasis is placed on daily seed consumption (linseeds, chia, hemp seeds, pumpkin), in part to obtain sufficient short-chain omega-3 fats. Extra virgin olive oil is included as part of the dietary plan. Patients are asked to avoid the following food additives and emulsifiers; carboxymethylcellulose, polysorbate 80 (P80), propylene glycol alginate, carrageenans, gums, (Arabic, Guar, xanthan), maltodextrin, glycerol monolaurate, locust bean gum, magnesium stearate, titanium dioxide, artificial colouring, hydrogenated oil (in supplements). Patients are advised to avoid alcohol consumption whilst they have symptoms and only consider reintroduction (if desired) at low levels once symptoms have resolved. Given that physical activity can be difficult for people with gastrointestinal symptoms a minimum of 15 minutes walking is recommended.

## Discussion

The three case histories presented, with more than 2 years of follow-up, highlight the potential role of a WFPBD in the treatment approach for people with UC. Given the central role of dietary risk factors in the development of UC, it makes sense to eliminate or greatly reduce the foods that are implicated, namely, meat, ultra-processed foods and exposure to food additives and emulsifiers.^9^ Standard treatment for UC involves remission induction followed by maintenance treatment to reduce the risk and frequency of flares.^12^ All three of our patients had failed to respond adequately to first-line treatment, namely, mesalazine. Second-line options at this stage would have been the addition of steroids or changing to a thiopurine or biologic therapy such as infliximab, adalimumab or vedolizumab. All of these medications suppress the immune system and therefore increase the risk of infection and potentially cancer.^13^ Treatment-free remission can be challenging to achieve, with most patients experiencing a relapse on discontinuation of therapy.^14,15^ Thus, a therapeutic dietary approach is an attractive option given the lack of side effects and the potential to limit medication use.

The use of plant-based diets in IBD has been pioneered by Japanese researchers who have used this in clinical practice for over 15 years, with remarkable results.^10,11,16-20^ Their plant-based diet is a semi-vegetarian diet that includes yoghurt and allows for fish once a week to make it more acceptable to patients. Their plant-based diet scoring system considers eight foods to be preventive factors for IBD (vegetables, fruits, pulses, potatoes, rice, miso soup, green tea, and plain yoghurt) and eight foods to be harmful (meat, minced or processed meat, cheese/butter/margarine, sweets, soft drinks, alcohol, bread and fish). They have achieved very good results and durable remissions in both UC and Crohn’s disease and have also demonstrated higher remission rates in combination with biological therapies. They have not found a negative impact with the high fibre content despite the prevailing advice to limit fibre consumption in the acute phase of the disease.

There are some interesting points to raise about the 3 cases presented. Constipation was a predominant symptom in case 1. This has been well described in UC and can be difficult to manage, with increasing fibre intake a key recommendation.^21^ Case 1 did initially have difficulty in tolerating an increase in fibre-rich foods, especially legumes, but a short duration of lowering exposure to FODMAPS with a more gradual increase in fibre helped support the transition to a WFPBD. Case 1 and 3, when returning to a meat-based diet, had recurrence of symptoms, but on returning to a predominantly WFPBD were able to achieve symptomatic remission. Case 1 and 2 have been able to avoid pharmaceutical medications altogether.

A plant-based diet addresses the key pathogenic mechanisms that are central to the development of IBD, namely, inflammation, increased intestinal permeability and gut dysbiosis.^22,23^ Fibre-rich plant-based diets improve the diversity and richness of the gut microbiota, leading to an increase in short-chain fatty acids, favourable changes in the immune cell profile and a reduction or resolution of gut inflammation. This has been demonstrated in a small cross-over study of 17 patients with UC in remission or with mild disease in which participants were randomly assigned to a low-fat high, fibre diet or the control diet (similar to the standard American diet) and blood and faecal samples analyses.^24^ The results showed that the high-fibre diet reduced markers of inflammation and improved gut microbial health.

Dietary guidance from the International Organisation for the Study of IBD supports the greater consumption of whole plant foods, whilst limiting red and processed red meat and ultra-processed foods, including food additives and emulsifiers.^25^ However, because of the lack of randomised controlled studies, the level of evidence supporting the recommendations for most of the foods and food groups reviewed is considered low, and overall recommendations remain rather moderate and in line with healthy eating guidelines. The consumption of fish remains a central recommendation due to the long-chain omega-3 fat content and the potential for anti-inflammatory effects. This is despite the authors acknowledging that the evidence supporting this recommendation remains weak. Our 3 patients were able to achieve remission without the consumption of fish. Omega-3 fats are essential to obtain from dietary sources and our patients obtained short-chain omega-3 fats from linseed, hemp seeds and chia seeds. Algae-sourced long-chain omega-3 fats are an option too.

Avoidance of certain food additives, in particular emulsifiers, is increasingly considered important for the prevention and treatment of IBD due to their ability to promote intestinal inflammation, alter gut permeability and the negative impact on gut microbiota.^26^ These compounds are found predominantly in ultra-processed foods, both animal- and plant-based products. Thus diet quality when adopting a plant-based diet remains paramount. Some plant-based dairy alternatives also contain emulsifiers and caution with these products in the setting of IBD may be a sensible approach. See Table 2 for a homemade almond milk recipe.

**Table 2.** Home Made Almond Milk Recipe by Despina Marselou.

Ingredients	Method
100 grams almonds, 1 litre of filtered water, 1 tablespoon of molasses and 4 dates or 50 grams chestnuts (if in season, boiled and without the shell)	1. Soak almonds (and chestnuts) in water in a large container overnight
	2. Discard soaking water (or use to water your plants!)
Optional: Can be fortified with calcium using a freeze dried calcium/magnesium complex or calcium made from Lithothamnium Calcereum (natural sea vegetable)	3. Blend the almonds, molasses and dates (or chustnuts) with the 1 litre of water
	5. Filter through a fine sieve lined with cheesecloth and squeeze tightly
	6. Store in an airtight bottle and use within 3 days

It is clear that most patients with IBD assume diet is an important factor in the development and treatment of the disease. Patients often report foods they consider to worsen their symptoms and most patients eliminate certain foods from their diet.^27,28^ Yet, most patients still do not receive adequate dietary counselling from their clinical team. This is despite the fact that there is sufficient evidence to support a plant-based dietary approach alongside conventional pharmaceutical treatment. In addition, dietary counselling is relevant in supporting better overall health in people living with IBD who have a significantly increased risk of cardiovascular disease,^29,30^ type 2 diabetes^31,32^ and certain cancers,^33^ when compared to the general population. A healthy diet alongside other healthy lifestyle behaviours has the ability to significantly reduce mortality in people with IBD.^34^

There are of course limitations with interpretation of such a small case series, which is merely hypothesis generating. Nonetheless, there is enough evidence to support a plant-based diet in other chronic conditions to suggest that this is also a viable option for patients with UC. In addition, our patients were supported to adopt other healthy lifestyle behaviours, which may also have positively impacted the favourable outcome.

In conclusion, this small case series highlights the therapeutic use of a WFPBD for remission induction and maintenance in UC, which may support the reduction or elimination of conventional pharmaceutical therapies.
